# The Effects of Children’s Smartphone Addiction on Sleep Duration: The Moderating Effects of Gender and Age

**DOI:** 10.3390/ijerph18115943

**Published:** 2021-06-01

**Authors:** Ji-Yeon Yoon, Kyu-Hyoung Jeong, Heeran J. Cho

**Affiliations:** 1 Institute for Life and Culture, Sogang University, Seoul 04100, Korea; y-research@naver.com; 2 Department of Social Welfare, Semyung University, Jecheon 27136, Korea; 3 Department of Health Administration, Yonsei University, Seoul 03021, Korea

**Keywords:** addictive behavior, adolescents, mental health, sleep duration, smartphone addiction

## Abstract

Background: Smartphones are an important part of children’s and adolescents’ lives, and they often spend a lot of time using them. This study aims to precisely discover the effects of smartphone addiction on sleep duration as moderated by age and gender. Materials and methods: The data utilized in this study are from the ‘Korean Children and Youth Panel Survey 2018′ by the National Youth Policy Institute; a total of 4940 youths (2399 in grade 4 and 2541 in grade 7) from the survey were analyzed by Stata 15.0 S. The dependent variable is sleep duration, and the independent variables are the sub-factors of smartphone addiction: disturbance of adaptive functions, virtual life orientation, withdrawal, and tolerance. An independent *t*-test was conducted to confirm the differences in the main variables according to gender and age. Multiple regression analysis was conducted to verify the moderating effects of gender and age in the relationship between children’s smartphone addiction and sleep duration. Results: First, the average sleep duration among grade 4 students was 9.17 h and grade 7 students was 7.96 h. Second, sleep duration was significantly higher for males than females, while there was no difference in smartphone addiction by gender. Third, smartphone addiction, particularly the sub-factor of tolerance significantly affected sleep duration. Fourth, age significantly affected sleep duration and gender had a moderating effect on sleep duration. Conclusions: Interventions to develop a healthy smartphone usage culture on family and societal levels would be beneficial for increasing awareness of smartphone addiction and its adverse effects on children and adolescents. Furthermore, targeted intervention would be more effective at modifying addictive behavior and sleep duration than trying to administer blanket interventions to youths as a whole.

## 1. Introduction

As mobile phones have become more advanced, offering users increasing capabilities and becoming more computer-like than cellphone-like, the new term “Smartphone” was adopted [1]. Smartphones allow access to a nearly limitless amount of education, knowledge, and every connected person in the world [2]. Smartphones are becoming increasingly like miniature laptops as they offer web browsing, WiFi, and a variety of educational and entertainment applications, and smartphones are popular and widely available [3]. Additionally, many modern smartphones have sleek and attractive designs, and their features are constantly evolving to be more convenient for users, especially for children and adolescents.

In Korea, 90% of 10- to 18-year-old children and adolescents owned a smartphone in 2018; this figure has been rapidly increasing since 2012 [4]. Korean children’s and adolescents’ average smartphone usage time is 36.2 h per week, which is 12.7 h longer than that of Korean adults’ usage [5]. It has been revealed that 3 out of 10 adolescents were shown to be over-dependent on smartphones, and those students used their smartphones for 63.5 h per week, or more than 9 h per day on average, negatively affecting their sleep and studies [5].

While smartphones have many productive applications, such as sending e-mails or utilizing reference and productivity applications [6], their overuse can cause a wide range of problems. Excessive smartphone use may be harmful to fingers, hands, wrists, and forearms [7]. Poor posture may cause damage to the muscles and bones of the neck and spine [8], and people may also be susceptible to neurological disorders such as depression [9,10,11,12]. It has also been reported that excessive use of smartphones leads to an increase in psychological problems, mainly among young people [13,14]. Turel and Serenko submitted that addictions may go beyond the scope of substance addictions, and smartphone addiction is an example of this [10].

According to Kim, excessive use of smartphones can be a sign of smartphone addiction [15], which is defined as compulsive and problematic behavioral patterns in using smartphones, inability to control use of smartphones, experiencing withdrawal symptoms when unable to use smartphones, increased tolerance with greater use, and functional impairment [16,17]. Scholars have attempted to define various sub-categories of smartphone addiction. Lin and her colleagues revealed and outlined the components of smartphone addiction as tolerance, withdrawal, compulsive symptoms, and functional impairment [16] and Kim outlined the components of smartphone addiction as tolerance, withdrawal, disturbance of adaptive functions, and virtual orientation [18].

Studies have reported contradictory findings regarding gender differences and smartphone addiction. Some revealed that females are more likely to be addicted to smartphones and engage in problematic smartphone usage [19,20,21,22,23,24,25]. Other studies showed that males experienced higher levels of problematic smartphone usage compared to females [9,26,27]. There are also studies that found no gender effect [28]. Age and smartphone usage have showed some degree of correlation. According to the Internet Addiction Survey (2011), smartphone addiction is more prevalent among teenagers than users in their twenties [29]. It has been noted that when people have a low level of education, they are more likely to be addicted to smartphones [29], and it is indicated that young children in general might be susceptible to smartphone addiction as they are too young to make rational decisions [30]. A recent study showed that 1 in 4 middle school students tended to be addicted to smartphones, and increasing numbers of elementary students have access to smartphones [31].

Smartphone use was shown to affect children and adolescents, causing them to have insufficient, disturbed or poor-quality sleep [32,33,34], which has emerged as a public health issue in technologically advanced societies [35]. Specifically, young people often tend to use their smartphones in their beds and before going to sleep [36], leading to insufficient sleep duration [37] and impaired sleep quality accordingly. It is also reported that excessive electronic media (e.g., smartphone) use at night is a risk factor for adolescents’ sleep disturbance and depression [38,39]. Likewise, previous studies reported that smartphone addiction negatively affect sleep duration which may cause adverse health issues.

While various studies have been conducted regarding smartphone addiction, few studies dealt with smartphone addiction sub-factors precisely in relation to gender and age using a large representative national sample. In this study, we investigated smartphone addiction as it affects sleep duration by means of its sub-factors, which include disturbance of adaptive functions, virtual life orientation, withdrawal, and tolerance in relation to gender and age, utilizing the ‘Korean Children and Youth Panel Survey 2018 (KCYPS 2018)’ from the National Youth Policy Institute (NYPI), in Sejong, South Korea.

## 2. Materials and Methods

### 2.1. Research Model

**Figure 1:**
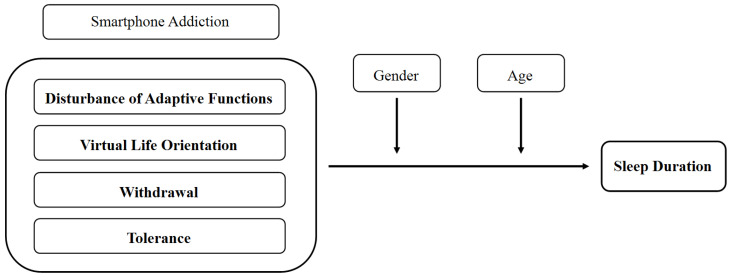
Research Model.

The research model was designed to examine the moderating effects of gender and age in the relationship between smartphone addiction and sleep duration in children and adolescents (Figure 1). The sub-factors of smartphone addiction—disturbance of adaptive functions, virtual life orientation, withdrawal, and tolerance—and their effects on sleep duration [17] were put forward in this study.

### 2.2. Data

The data utilized in this study are from the KCYPS 2018, which is the most recent available survey from the NYPI. The Korean Children and Youth Panel Survey is a nationwide representative survey of children and adolescents in Korea. It aims to produce basic data to be used to establish effective child and youth policies by comprehensively grasping various aspects of child and adolescent growth and development. As of 2018, the sample of KCYPS was extracted using a multi-stage cluster sampling method based on the grade 4 population, who were mostly born in 2008, and the grade 7 population, who were mostly born in 2005. A total of 5197 surveys were completed (2607 grade 4 students, 2590 grade 7 students). NYPI collected the data for only grades 4 and 7 to establish KCYPS 2018, which comprehensively grasp changes in the growth and development of children and adolescents; grade 4 to represent children and grade 7 to represent adolescents. In this study, 4940 youths (2399 in grade 4 and 2541 in grade 7) which had no missing values were included in the analysis. Regarding adequacy of the sample size, the minimum sample size was checked using G*Power 3 (G*Power, Düsseldorf, Germany), Effect Size f^2^ was set to 0.15, α error probability was set to 0.05, power (1-β probability) was set to 0.8 [40]. The results showed that a minimum value of 98 participants would be sufficient.

### 2.3. Measures

#### 2.3.1. Dependent Variable: Sleep Duration

The dependent variable of this study was sleep duration, which was based on average wake-up time and average bedtime. It is calculated as follows: (average wake-up time + 24) − average bedtime = sleep duration.

#### 2.3.2. Independent Variable: Smartphone Addiction

The independent variable of this study was smartphone addiction. The scale was modified from the Smartphone Addiction Proneness Scale (SAPS) developed by Kim and his colleagues [17] at the NYPI for children and adolescents (Table 1). SAPS consists of a total of 15 questions (5 disturbance of adaptive functions, 2 virtual world orientation, 4 withdrawal, 4 tolerance), and a 4-point scale (1 = strongly disagree, 2 = disagree, 3 = agree, 4 = strongly agree). Disturbance of adaptive functions consisted of 5 items including ‘my school grades dropped due to excessive smartphone use’ and ‘people frequently comment on my excessive smartphone use’. Virtual life orientation consisted of 2 items including ‘when I cannot use a smartphone, I feel like I have lost the entire world’. Withdrawal consisted of 4 items including ‘I get restless and nervous when I am without a smartphone’, and tolerance consisted of 4 items including ‘I try cutting my smartphone usage time, but I fail’. Out of 15 questions, ‘My smartphone does not distract me from my studies’, ‘I am not anxious even when I am without a smartphone’, ‘I don’t spend a lot of time using smartphone’ were coded in reverse. The average of the questions was calculated. The higher the score, the higher the level of smartphone addiction was interpreted. In this study, tolerance means spending increasing time on one’s smartphone, in the same way this concept was used in Lin et al. [16] and American Psychiatric Association [41]. Cronbach’s alpha was 0.884 and reliability was found to be reasonably high.

#### 2.3.3. Moderator Variable

The moderator variables used in this study are gender (male = 0, female = 1) and age (4th grade in elementary school = 0, 7th grade in middle school = 1).

### 2.4. Statistical Analyses

Stata 15.0 SE was used for the analysis. An independent *t*-test was conducted to confirm the difference in the main variables according to gender and age. Multiple regression analysis was conducted to verify the moderating effects of gender and age in the relationship between children’s smartphone addiction and sleep duration.

## 3. Results

### 3.1. Differences in the Main Variables According to Gender and Age

The results of the independent *t*-test to find out the differences of the main variables according to gender and age are shown in Table 2 and Table 3. Smartphone addiction did not show any statistically significant difference according to gender, and the sub-factors (the disturbance of adaptive functions, virtual life orientation, withdrawal, and tolerance) were also found to be statistically insignificant. However, regarding sleep duration, the males’ average was 8.64 h (SD = 1.01) and the females’ average was 8.45 h (SD = 1.15), and this difference was statistically significant (*t* = 6.342, *p* < 0.001). In the case of age, there was a significant difference in smartphone addiction and sleep duration. Specifically, smartphone addiction was significantly lower in grade 4 students (M = 1.75, SD = 0.50) than grade 7 students (M = 1.97, SD = 0.48) (*t* = −16.352, *p* < 0.001). Sub-factors, disturbance of adaptive functions, virtual life orientation, withdrawal, and tolerance were also found to be significantly lower in grade 4 students. The average sleep duration was 9.17 h (SD = 0.81) in grade 4 students and 7.96 h (SD = 0.97) in grade 7 students, and it was confirmed that the grade 4 students had a significantly longer sleep duration than the grade 7 students (*t* = 47.772, *p* < 0.001).

### 3.2. Analysis of Research Model

Table 4 shows the moderating effect of gender in the relationship between smartphone addiction and sleep duration. First, the coefficient of determination for sleep duration was 33.6% (R^2^ = 0.336), and the regression equation is statistically significant (F = 499.61, *p* < 0.001). The independent variable, smartphone addiction (B = −0.109, *p* < 0.05) and the moderator variable, age (B = −1.202, *p* < 0.001) had a significant effect on sleep duration. The lower the level of smartphone addiction, the longer the sleep duration was for grade 4 students compared to grade 7 students. Meanwhile, gender did not significantly affect sleep duration. For the interactions, smartphone addiction × gender (B = −0.177, *p* < 0.001) had a significant effect on sleep duration, but smartphone addiction × age was found to have no significant effect on sleep duration.

**Figure 2:**
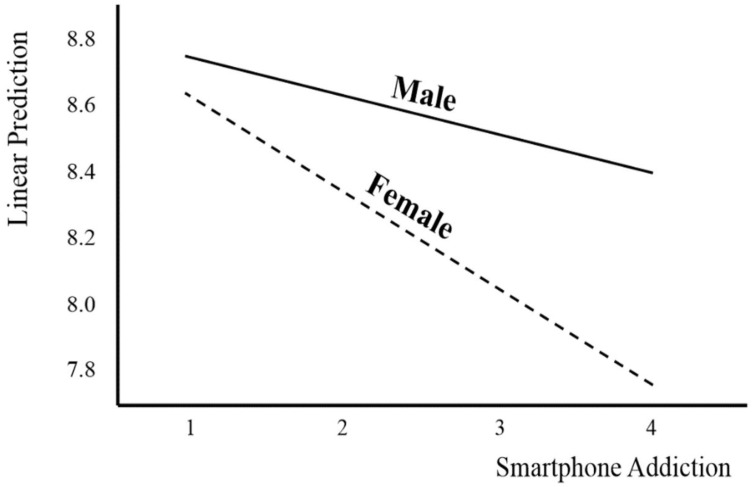
Moderating effect analysis result 1.

The effect of using gender as a moderating variable in the relationship between smartphone addiction and sleep duration is shown in Figure 2. It shows that the higher the level of smartphone addiction, the more rapidly the sleep duration for females decreased compared with males.

The effects of gender and age on the relationship between the sub-factors of smartphone addiction (disturbance of adaptive functions, virtual life orientation, withdrawal, and tolerance) and sleep duration are shown in Table 5. The coefficient of determination for sleep duration was 33.8% (R^2^ = 0.338), and the regression equation was statistically significant (F = 179.89, *p* < 0.001). Sub-factors of smartphone addiction, tolerance (B = −0.139, *p* < 0.01), as an independent variable, and age (B = −1.230, *p* < 0.001) as a moderator variable significantly affected sleep duration. In other words, the lower the tolerance score, the longer the sleep duration in grade 4 compared with grade 7, which means that grade 4 students had lower tolerance to smartphone use and got more sleep. Meanwhile, disturbance of adaptive functions, virtual life orientation, withdrawal, and gender did not significantly affect sleep duration. In the case of the interactions, only withdrawal × gender (B = −0.127, *p* < *0*.05) was found to have a significant effect on sleep duration.

**Figure 3:**
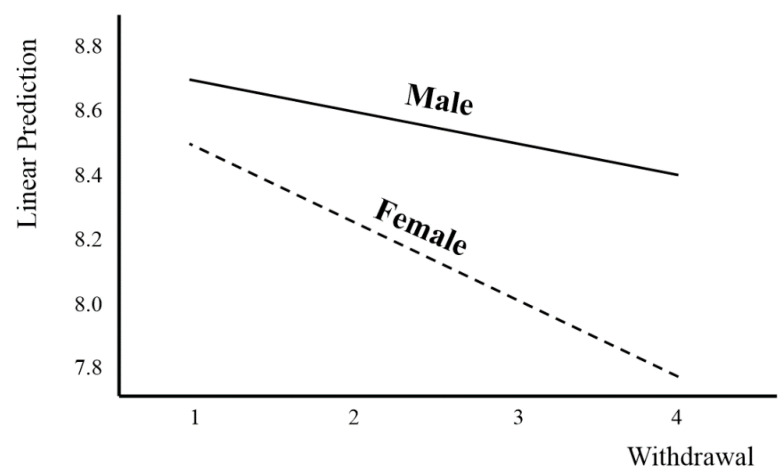
Moderating effect analysis result 2.

The moderator effect of gender in the relationship between withdrawal and sleep duration is shown in Figure 3. It shows that the higher the degree of withdrawal was, the more rapidly the sleep duration decreased in females (Figure 3).

## 4. Discussions

This study examined the relationship between smartphone addiction and sleep duration with moderating effects of gender and age for the 4940 participants in grade 4 and grade 7 living in South Korea, based on the KCYPS 2018. Specifically, the sub-factors of smartphone addiction—disturbance of adaptive functions, virtual life orientation, withdrawal, and tolerance—were investigated in relation to sleep duration. Detailed research discussion points are as follows.

Firstly, there was no difference in smartphone addiction by gender, but sleep duration was significantly longer for males than females. While previous studies showed contradictory results regarding gender [19,20,21,22,23,24,25,26,27,28], this study showed that gender had no significant effect on smartphone addiction, corroborating the studies of Demirci et al. [28]. Furthermore, there were significant differences for smartphone addiction and sleep duration between grade 4 and grade 7 students. Grade 7 students were more addicted to smartphones and had a shorter sleep duration than grade 4 students; grade 7 students experienced more disturbance of adaptive functions, and ranked more highly on measures of virtual life orientation, withdrawal, and tolerance to smartphones than grade 4 students. Park and Park [42] indicated that children could easily become addicted to smartphones because they have not reached the age when they are able to make rational decisions. However, this study suggests that although children still may have not reached an age when they are capable of making rational decisions, smartphone addiction could be more severe among young adolescents than children, as grade 4 students measured lower on smartphone addiction than grade 7 students.

Secondly, smartphone addiction significantly affected sleep duration; in particular, sleep duration was affected by increased tolerance scores meaning that though students tried to control their smartphone use, they were more likely to fail, leading to shorter sleep duration. It was shown that grade 7 students were higher in tolerance, and their sleep duration was shorter than students in grade 4 accordingly. This also shows that students in higher grades, who are higher in tolerance to smartphones get less sleep, and it indicates that tolerance is an important sub-factor to measure smartphone addiction. According to Deniz, when smartphone dependency increases, there is a decrease in sleep duration, and those who are older are at greater risk of smartphone addiction [43]. It was also reported that excessive use of smartphones causes depression and/or anxiety, which may lead to sleep problems [44]. A study conducted in Belgium suggested that one quarter of young people with smartphones experience disturbed sleep patterns [45]. In South Korea, Kim showed that sub-factors of smartphone addiction such as daily life problems, withdrawal symptoms, and loss of control are related to insufficient sleep [46].

Thirdly, gender has a moderating effect in the relationship between withdrawal from smartphones and sleep duration; the higher the degree of withdrawal was, the more rapidly the sleep duration decreased in females. This result is in line with the studies reporting that females are more likely to engage in problematic smartphone use [18,19,20,21,22,23,24]. It was also shown in a study conducted in India that smartphone usage was higher for females than males [47]. Furthermore, it was found that a health-promoting lifestyle positively associated with sleep duration and smartphone dependence was a significant predictor of sleep deprivation for female juniors in college [48].

On the other hand, this study analyzed grade 4 and grade 7 students in general, and did not focus specifically on students who are severely addicted to smartphones, so the mean scores of smartphone addiction and its sub-factors are mostly below 2 (Table 2) and the average sleep duration of grade 4 students (9.17 h) and grade 7 students (7.96 h) might not look so problematic. However, as smartphone addiction among children and adolescents is becoming more severe, resulting in increased sleep deprivation among those who are over-dependent on smartphones [5,6], the significant results of this study should be considered seriously.

## 5. Conclusions

This study is meaningful as it explored the effects of the sub-factors of smartphone addiction on sleep duration in greater depth in relation to gender and age among children and adolescents from a large set of representative national sample data. As smartphone addiction and insufficient sleep affect health adversely, considering the insight of this study would be beneficial.

This study presented the relationships between smartphone addiction and sleep duration with the moderating effects of age and gender, and shed light on tolerance as a main factor that decreases sleep duration for adolescents, and withdrawal as a main factor that affects the sleep duration of females. This study suggests that interventions would be beneficial to prevent smartphone addiction among youth of different ages and genders. It would be more efficient to carry out separate programs for different gender and age groups, considering smartphone addiction sub-factors like tolerance and withdrawal. The behaviors and thought processes that would manifest due to these sub-factors at different stages of development and in different genders would likely require highly targeted and specialized interventions to be resolved successfully.

Specifically, it would be efficient and prudent for schools, society, and governments to provide healthy smartphone usage culture education not only to children and adolescents, but also their parents. In this way, children and adolescents can become aware of the severity of smartphone addiction and its adverse effects at the family and societal levels. Furthermore, when implementing programs for children and adolescents, it may be necessary to focus more on females, as females are more prone to smartphone addiction than males. Smartphone addiction rehab would be also helpful for students who have sleeping problems, providing treatment in more specialized ways. Likewise, more targeted intervention would be more essential for modifying addictive behavior and sleep duration, instead of targeting youth as a whole.

This study had the following limitations and recommendations for future studies. First, as we used secondary data, there was a limitation in the original data, and the students’ ages were restricted to grades 4 and grade 7. More accurate results regarding age could be determined if there were more available data covering a more diverse set of ages. In future studies, collecting data for additional age groups would result in more precise assessments. Second, this study looked at grades 4 and grade 7 without focusing on students who were seriously addicted to smartphones. In order to understand the seriousness of smartphone addiction and sleep duration in depth, focusing on the group which has been most impacted would be efficient for future studies.

## Figures and Tables

**Table 1:** Smartphone Addiction Proneness Scale for Children and Adolescents.

Subdomain	Items
Disturbance of Adaptive Functions	1. My school grades dropped due to excessive smartphone use
	2. I have a hard time doing what I have planned (study, do homework, or go to afterschool classes) due to using smartphone
	3. People frequently comment on my excessive smartphone use
	4. Family or friends complain that I use my smartphone too much
	5. My smartphone does not distract me from my studies
Virtual world orientation	6. Using a smartphone is more enjoyable than spending time with family or friends
	7. When I cannot use a smartphone, I feel like I have lost the entire world
Withdrawal	8. It would be painful if I am not allowed to use a smartphone
	9. I get restless and nervous when I am without a smartphone
	10. I am not anxious even when I am without a smartphone
	11. I panic when I cannot use my smartphone
Tolerance	12. I try cutting my smartphone usage time, but I fail
	13. Even when I think I should stop, I continue to use my smartphone too much
	14. Spending a lot of time on my smartphone has become a habit
	15. I don’t spend a lot of time using smartphone

**Table 2:** Differences in the main variables by gender.

Variables	Male (*n* = 2568)		Female (*n* = 2372)		*t*
	M	SD	M	SD	
Smartphone addiction	1.87	0.50	1.86	0.51	0.418
Disturbance of adaptive functions	2.11	0.60	2.08	0.62	1.723
Virtual life orientation	1.52	0.61	1.50	0.59	0.873
Withdrawal	1.64	0.57	1.64	0.58	0.020
Tolerance	2.20	0.67	2.22	0.72	−1.058
Sleep duration	8.64	1.01	8.45	1.15	6.342 ***

Note: *n* = sample size, M = mean, SD = standard deviation, *t* = *t*-value, *** *p* < 0.001.

**Table 3:** Differences in the main variables by age.

Variables	Grade 4 (*n* = 2399)		Grade 7 (*n* = 2541)		*t*
	M	SD	M	SD	
Smartphone addiction	1.75	0.50	1.97	0.48	−16.352 ***
Disturbance of adaptive functions	1.97	0.60	2.22	0.58	−14.511 ***
Virtual life orientation	1.41	0.57	1.61	0.61	−11.933 ***
Withdrawal symptoms	1.54	0.56	1.73	0.58	−11.524 ***
Tolerance	2.06	0.70	2.34	0.66	−14.407 ***
Sleep duration	9.17	0.81	7.96	0.97	47.772 ***

Note: *n* = sample size, M = mean, SD = standard deviation, *t* = *t*-value, *** *p* < 0.001.

**Table 4:** Research model analysis 1.

Variables		B	S.E.
Independent variable	Smartphone Addiction (A)	−0.109 *	0.044
Moderator variable	Gender (B)	0.082	0.097
	Age (C)	−1.202 ***	0.099
Interaction	A × B	−0.177 ***	0.050
	A × C	0.016	0.051
Constant		9.473	0.082
R^2^		0.336	
F(sig.)		499.61 ***	

Note: B = B-static, S.E = standard error, sig. = significance * *p* < 0.05, *** *p* < 0.001.

**Table 5:** Research model analysis 2.

Variables		B	S.E.
Independent variable	Disturbance of adaptive functions (A)	−0.048	0.050
	Virtual life orientation (B)	0.008	0.051
	Withdrawl (C)	0.052	0.055
	Tolerance(D)	−0.139 **	0.042
Moderator variable	Gender (E)	0.064	0.099
	Age (F)	−1.230 ***	0.102
Interaction	A × E	−0.027	0.058
	B × E	−0.066	0.059
	C × E	−0.127 *	0.069
	D × E	−0.028	0.051
	A × F	0.082	0.058
	B × F	−0.087	0.059
	C × F	0.017	0.063
	D × F	−0.004	0.051
Constant		9.493	0.084
R^2^		0.338	
F(sig.)		179.89 ***	

Note: B = B-static, S.E = standard error, sig. = significance * *p* < 0.05, ** *p* < 0.01, *** *p* < 0.001.

## Data Availability

The data presented in this study are available in https://www.nypi.re.kr/archive/board?menuId=MENU00252.
